# Triptolide induces protective autophagy through activation of the CaMKKβ-AMPK signaling pathway in prostate cancer cells

**DOI:** 10.18632/oncotarget.6783

**Published:** 2015-12-29

**Authors:** Fei Zhao, Weiwei Huang, Zhe Zhang, Lin Mao, Yangyang Han, Jun Yan, Ming Lei

**Affiliations:** ^1^ College of Life Sciences, Northwest A & F University, Yangling, Shaanxi Province, People's Republic of China; ^2^ Institute of Biophysics, Chinese Academy of Sciences, Chaoyang District, Beijing, People's Republic of China; ^3^ State Key Laboratory of Pharmaceutical Biotechnology and MOE Key Laboratory of Model Animals for Disease Study, Model Animal Research Center of Nanjing University, Nanjing, Jiangsu Province, People's Republic of China

**Keywords:** triptolide, CaMKKβ, AMPK, autophagy, apoptosis

## Abstract

Triptolide, an active compound extracted from the Chinese herb thunder god vine (*Tripterygium wilfordii* Hook F.), has potent anti-tumor activity. Recently, triptolide was found to induce autophagy in cancer cells. However, the effects of triptolide on autophagy in human prostate cancer (PCa) cells have not yet been clearly elucidated. In this study, we demonstrated that triptolide induces autophagy in three PCa cell lines, PC-3, LNCaP and C4–2. Furthermore, we found that triptolide mediates intracellular accumulation of free calcium by stimulating the endoplasmic reticulum (ER) stress response. This activates the CaMKKβ-AMPK signaling pathway, which in turn inhibits mTOR and activates both ULK1 and Beclin 1, finally resulting in autophagy. Moreover, we found that treatment with autophagy inhibitors 3-methyladenine (3-MA) and chloroquine (CQ) enhances triptolide-induced PCa cell death and growth inhibition. Using a PC-3-xenografted mouse model, we showed that blocking autophagy with CQ significantly promoted triptolide-induced tumor growth inhibition *in vivo*. Overall, our results show that triptolide induces protective autophagy through the CaMKKβ-AMPK pathway in PCa cells, implying that a combination of triptolide with autophagy inhibitors may potentially be an effective therapeutic strategy for PCa.

## INTRODUCTION

Triptolide, a diterpene triepoxide, is the major active compound extracted from a traditional Chinese medicinal herb named thunder god vine (*Tripterygium wilfordii* Hook F.), which is mainly used to treat autoimmune diseases including rheumatoid arthritis, psoriasis and lupus. Beyond the immunosuppression effect, triptolide shows many other pharmacological activities, such as reduction of inflammation, contraceptive activity, decreased cyst formation, and so on [1]. Triptolide also shows potent anti-tumor effects, which has attracted much attention and has been studied intensively. Many studies have demonstrated that triptolide has broad-spectrum anti-tumor efficacy. Triptolide shows anti-tumor effects on almost all kinds of cancer cell *in vitro* and *in vivo*, including breast cancer, lung cancer, pancreatic cancer, colon cancer, thyroid cancer, leukemia, and so on [1–4]. Our previous studies demonstrated that triptolide has effective anti-PCa and anti-laryngocarcinoma activity [5, 6]. Triptolide also exhibits very high anti-tumor activity. Titov et al. reported that triptolide inhibits the proliferation of all 60 cancer cell lines from the US National Cancer Institute with an average IC_50_ of just 12 nM [7].

The anti-tumor effect of triptolide is mainly due to its induction of cancer cell death. As the major cause of cell death, triptolide-induced apoptosis has been well studied. It has been proven that triptolide-induced apoptosis in cancer cells shows typical apoptotic characteristics, such as nuclear fragmentation, DNA damage, cytochrome C release, caspase activation, PARP1 cleavage, and changes in the expression of apoptotic proteins [1]. In addition to the apoptosis induction effect, recent studies showed that triptolide also shows anti-tumor effect on pancreatic cancer and neuroblastoma cells through induction of autophagy [8, 9]. However, the mechanism that underlies the induction of autophagy by triptolide is still unclear, especially in PCa.

Autophagy is a highly conserved catabolic mechanism in eukaryotes through which intracellular contents including large protein complexes and dysfunctional organelles are packaged and transported to lysosomes for degradation and re-use [10]. Three forms of autophagy are commonly referred to, namely macroautophagy, microautophagy and chaperone-mediated autophagy, among which macroautophagy (called autophagy in this paper) is the best understood [10]. As a basic catabolic mechanism, autophagy degrades and recycles unnecessary or dysfunctional cellular components, thus maintaining intracellular homeostasis, preventing cumulative cellular damage and cell aging, and promoting the cellular survival response to nutrient starvation and stresses [11]. Extensive autophagy may lead to cell death which is defined as autophagic programmed cell death (PCD) [10, 12]. Autophagy is also involved in pathological processes including neurodegenerative diseases, infection and cancer [13–15]. The role of autophagy in cancer has drawn the most attention, because it may provide a viable option for cancer therapy. Previous research suggested that autophagy acts as a double-edged sword in cancer survival by playing opposing roles of protector and inhibitor according to the cancer type or grade [12, 14, 16]. On the one hand, by maintaining homeostasis, autophagy recycles cellular components and promotes energy production to meet the high metabolic demands of cancer cells, thus supporting cancer cell survival. On the other hand, autophagy reduces cell instability and damage to prevent tumorigenesis. Until now, many anti-tumor compounds including triptolide have been found to have an autophagy-inducing effect, in spite of the fact that autophagy is associated with drug resistance in some cases [17, 18].

In the present study, we investigated the autophagy induction effect of triptolide on PCa cells, and examined the underlying molecular mechanisms. Our results demonstrate that triptolide induces autophagy in PC-3, LNCaP and C4–2 cells. Further studies showed that triptolide activates the AMPK pathway, which in turn inhibits mTOR and activates ULK1 and Beclin 1, thus promoting autophagy. AMPK activation is mainly due to CaMKKβ-mediated phosphorylation of AMPKα Thr172 in response to accumulation of free calcium in the cytoplasm. This calcium is released from the ER as a result of triptolide-induced ER stress. Furthermore, we found that autophagy plays a cytoprotective role in PCa cells, defending the cells against triptolide cytotoxicity *in vitro* and *in vivo*. Overall, our findings suggest that triptolide induces protective autophagy in PCa cells through the CaMKKβ-AMPK signaling pathway. This let us to anticipate that a combination of triptolide and autophagy inhibitors may be a more effective therapeutic strategy for treating prostate cancer.

## RESULTS

### Triptolide induces autophagy in PCa cells

**Figure 1:**
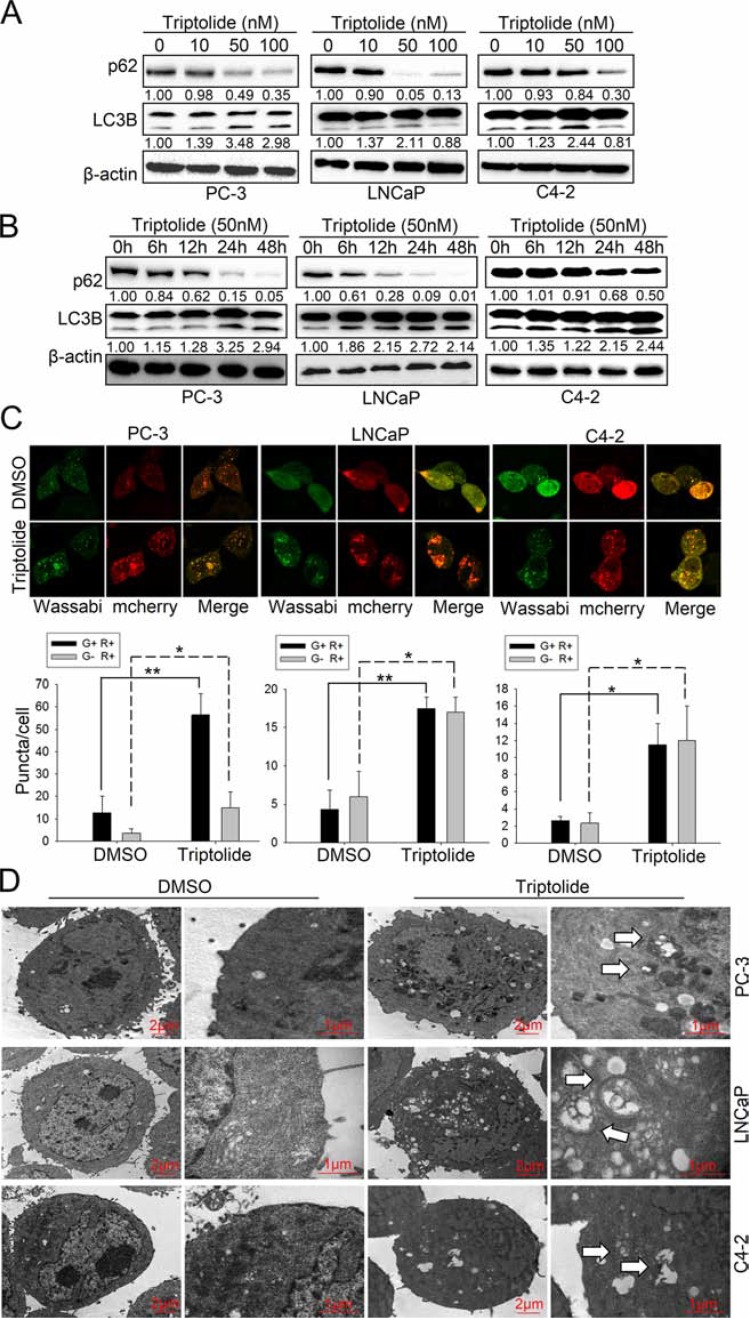
Triptolide induces autophagy in PCa cells (**A**) PC-3, LNCaP and C4–2 cells were treated with the indicated concentrations of triptolide and incubated for 24 h. Levels of protein expression were analyzed by western blot using antibodies against LC3B, p62 and β-actin. The number under each band represents the fold change of band intensity relative to control group. The fold change of LC3B only refers to LC3B II. (**B**) PC-3, LNCaP and C4–2 cells were treated with 50 nM triptolide for the indicated times. Levels of protein expression were analyzed by western blot using antibodies against LC3B, p62 and β-actin. (**C**) PC-3, LNCaP and C4–2 cells stably transfected with mCherry-Wassabi-LC3B were treated with 50 nM triptolide for 24 h, then subjected to confocal microscope analysis. The graphs show the average number of two kinds of LC3B puncta per cell. Green-positive, red-positive puncta (G+R+) are autophagosomes; green-negative, red positive puncta (G–R+) are autophagolysosomes. (**D**) PC-3, LNCaP and C4–2 cells were treated with DMSO or 50 nM triptolide for 24 h, then harvested and subjected to transmission electron microscopy analysis. The right panels show enlarged regions of the left panels. The arrows indicate autophagosomes. “*P < 0.05. **P < 0.01.

To determine whether triptolide induces autophagy in PCa cells, we used three human prostate cancer cell lines, PC-3, LNCaP and C4–2, as models and treated with triptolide. We first examined LC3B-II formation and the level of p62 in PCa cells treated with triptolide. After treatment with triptolide at the indicated concentration for 24 h (Figure 1A), or with 50 nM triptolide for the indicated time (Figure 1B), LC3B-II levels were increased in PC-3, LNCaP and C4–2 cells in both dose-dependent and time-dependent manners. Meanwhile, the p62 level decreased in similar manners. These results indicate that triptolide may induce autophagy in PCa cells. We also monitored the formation of autophagosomes in these PCa cell lines stably transfected with tandem mCherry-Wassabi-LC3B. In these cells, early autophagosomes display both green signal (Wassabi) and red signal (mCherry). Autophagolysosome display only red fluorescence since the Wassabi signal is sensitive to the acidic conditions in the lysosome lumen while the mCherry signal is more stable. After cells treatment with triptolide, autophagosomes were detected by confocal microscopy. The number of yellow puncta (both green and red positive, G+R+) and red only puncta (G−R+) increased significantly in cells treated with 50 nM triptolide compared with untreated control cells (Figure 1C). Furthermore, more autophagic vesicles containing engulfed organelles were detected by transmission electron microscopy in triptolide-treated cells (Figure 1D). These results prove that triptolide induces autophagy in PCa cells.

**Figure 2:**
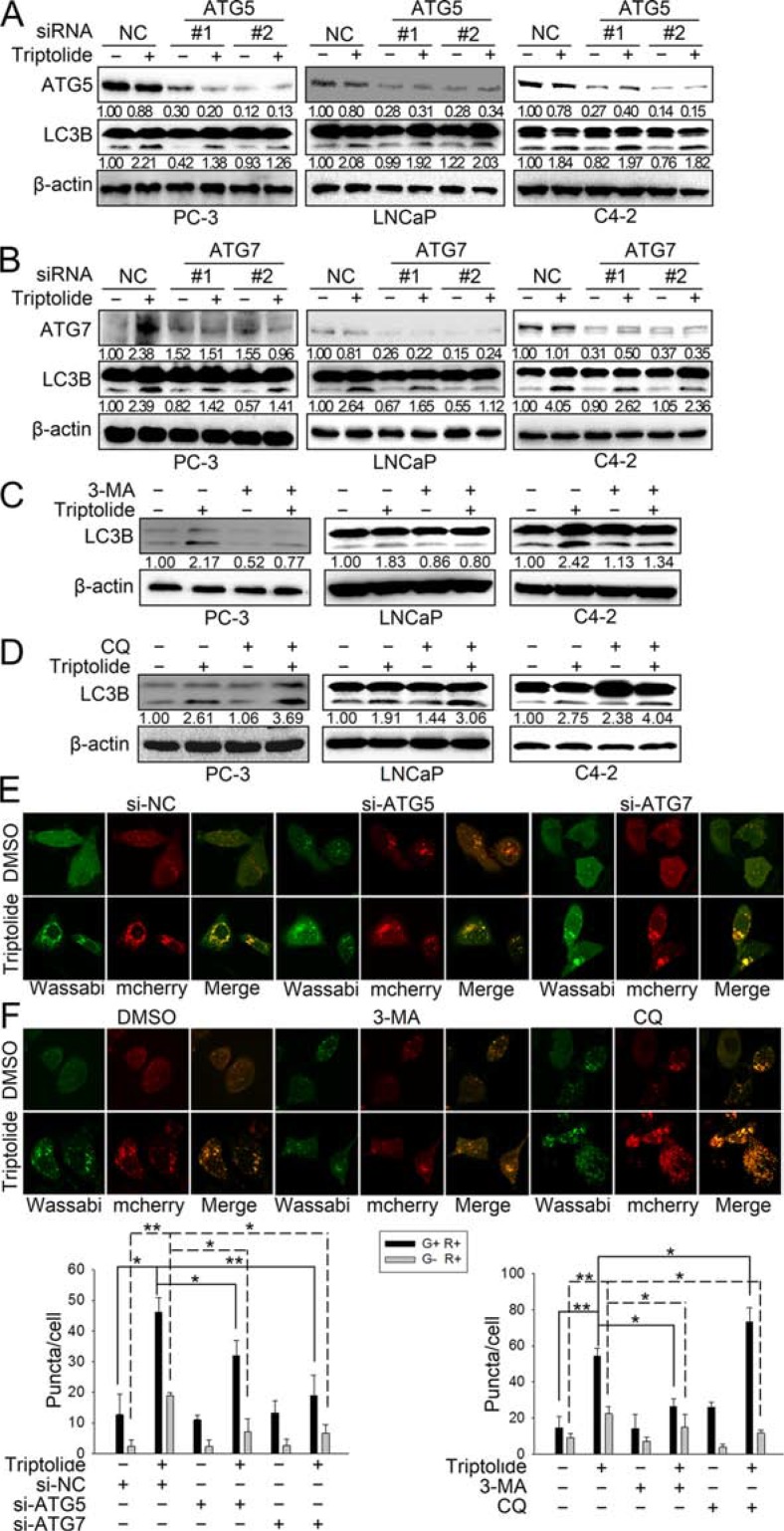
Triptolide induces autophagic flux in PCa cells (**A**) and (**B**) PC-3, LNCaP and C4–2 cells were transfected with ATG5 or ATG7 siRNAs for 24 h, and treated with 50 nM triptolide or DMSO for another 24 h. The samples were subjected to western blot analysis. The number under each band represents the fold change of band intensity relative to control group. (**C**) and (**D**) PC-3, LNCaP and C4–2 cells were pretreated with 3-MA (10 mM) or CQ (3 μM) for 1 h, and co-incubated with triptolide (50 nM) for another 24 h. Levels of protein expression were analyzed by western blot using antibodies against LC3B and β-actin. (**E**) PC-3 cells stably transfected with mCherry-Wassabi-LC3B were further transfected with ATG5 or ATG7 siRNAs, then treated with 50 nM triptolide or DMSO as mentioned above. The cells were subjected to confocal microscopy. (**F**) PC-3 cells stably transfected with mCherry-Wassabi-LC3B were co-treated with autophagy inhibitors and triptolide as mentioned above, then the cells were examined by confocal microscopy. The graphs display the average number of two different kinds of LC3B puncta per cell in the experiments show in (E) and (F). G+R+, autophagosomes; G−R+, autophagolysosomes. **P < 0.05. **P < 0.01.

Since autophagy is a dynamic and multi-step process, we continued to monitor the autophagic flux induced by triptolide (50 nM). As shown in Figure 2A, knockdown of ATG5 by siRNA decreased triptolide-induced LC3B-II formation in PC-3 cells, but showed no obvious effect on LC3B-II formation in LNCaP and C4–2 cells. This suggests that triptolide may induce autophagy in LNCaP and C4–2 cells in an ATG5-independent manner. Knockdown of ATG7 by siRNAs decreased triptolide-induced LC3B-II formation in all three cell lines (Figure 2B). Meanwhile, we performed similar experiments in the presence of the autophagy inhibitors 3-MA and CQ. 3-MA is a class III PI3-kinase inhibitor that blocks autophagy at an upstream step to decrease the production of LC3B-II. CQ is a lysosomotropic agent which prevents endosomal acidification, thus inhibiting lysosomal enzymes and causing accumulation of LC3B-II. The results showed that 3-MA (10 mM) reduced triptolide-induced LC3B-II formation in PCa cells (Figure 2C), while CQ (3 μM) increased LC3B-II accumulation (Figure 2D). We also examined the effect of knockdown of ATG5/7 or autophagy inhibitors on triptolide-induced LC3B puncta formation in PC-3 cells using confocal microscopy. As shown in Figure 2E and 2F, knockdown of ATG5 and ATG7 both decreased triptolide-induced LC3B puncta formation (Figure 2E). 3-MA reduced the number of both yellow spots (autophagosomes) and red spots (autophagolysosomes), indicating that 3-MA inhibited triptolide-induced autophagosome formation (Figure 2F). CQ increased the number of yellow spots and reduced the number of red spots, indicating that CQ induced accumulation of autophagosomes while reducing the formation of autophagolysosomes (Figure 2F). Overall, these data further confirm that triptolide induces autophagy in PCa cells.

### Triptolide induces autophagy by inhibiting the mTORC1 complex and activating both the ULK complex and the class III PI3K complex in PCa cells

**Figure 3:**
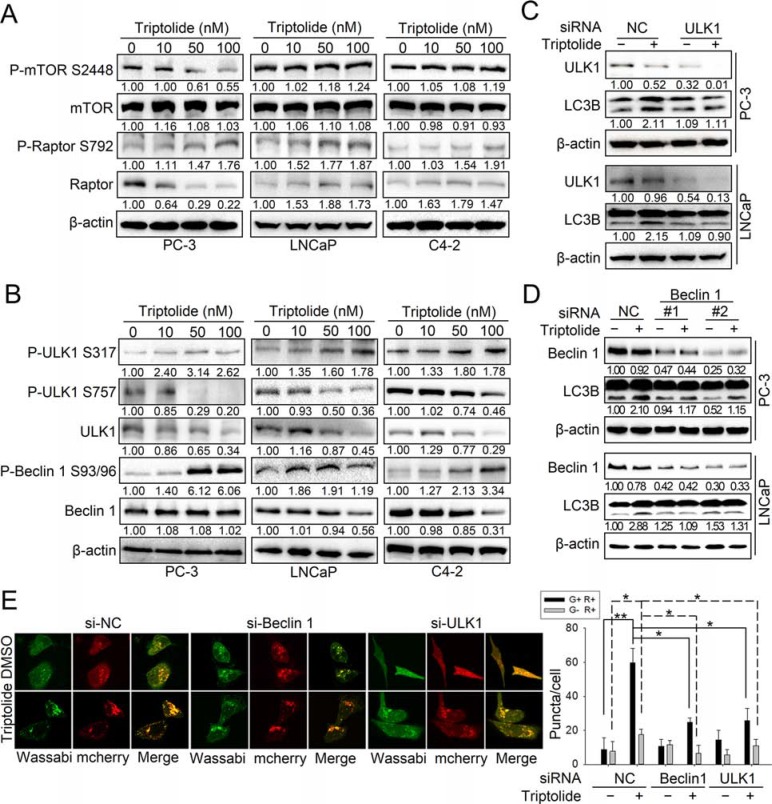
Triptolide inhibits the mTORC1 complex and activates both the ULK1 complex and the class III PI3K complex in PCa cells (**A**) and (**B**) PC-3, LNCaP and C4–2 cells were incubated with the indicated concentrations of triptolide for 24 h. Levels of protein expression were analyzed by western blot using antibodies against the indicated autophagy-related proteins and β-actin. The number under each band represents the fold change of band intensity relative to control group. (**C**) PC-3 and LNCaP cells were transfected with ULK1 siRNA or control (NC) siRNA for 24 h, then treated with 50 nM triptolide for another 24 h. Levels of protein expression were analyzed by western blot using antibodies against LC3B and β-actin. (**D**) PC-3 and LNCaP cells were transfected with Beclin 1 siRNAs or control (NC) siRNA for 24 h, then treated with 50 nM triptolide for another 24 h. Levels of protein expression were analyzed by western blot using antibodies against LC3B and β-actin. (**E**) PC-3 cells stably transfected with mCherry-Wassabi-LC3B were further transfected with ULK1 or Beclin1 siRNAs for 24 h, then treated with 50 nM triptolide for another 24 h. The cells were then examined by confocal microscopy. The average number of G+R+ and G−R+ LC3B puncta per cell is shown in the right chart. **P < 0.05. **P < 0.01.

We further examined the molecular mechanism underlying triptolide-induced autophagy. Autophagy is strictly regulated by multiple signaling pathways, in which mTOR acts as a critical negative-regulator [19]. Therefore, we first examined the effect of triptolide on the mTORC1 complex. As shown in Figure 3A, triptolide does not significantly affect the protein level of mTOR in three PCa cell lines. However, the level of P-mTOR Ser2448 was decreased by triptolide in PC-3 cells, but was not significantly changed in LNCaP and C4–2 cells. This implies that triptolide disrupts the activity of mTORC1 by directly suppressing the phosphorylation of mTOR at Ser2448 in PC-3 cells. Moreover, we tested the effect of triptolide on the regulatory associated protein of mTOR (raptor), an important subunit of mTORC1. Raptor acts as a binding partner of mTOR, and promotes the mTOR-mediated phosphorylation of p70S6k and 4E-BP1 [20]. Phosphorylation of raptor at Ser792 by AMPK induces 14–3–3 binding, which results in inhibition of mTOR kinase activity and induction of autophagy [20–22]. Our results showed that triptolide increases the level of P-raptor Ser792, but has different effects on total raptor in all three PCa cell lines (Figure 3A). This suggests that triptolide also indirectly inhibits mTOR activity by promoting raptor phosphorylation in PCa cells, and further implies that AMPK may be involved in triptolide-induced autophagy.

The autophagy pathway is strictly regulated by a series of ATG proteins, among which the ULK1 complex is essential for initiation of autophagy [23]. When nutrients are deficient, ULK1 is phosphorylated at Ser317, Ser555, and Ser777 by activated AMPK, leading to autophagy activation; in contrast, when nutrients are sufficient, ULK1 is phosphorylated at Ser757 by mTOR, leading to the disassociation of ULK1 and AMPK, which inhibits autophagy [24]. We therefore monitored the effect of triptolide on ULK1. We found that the protein level of ULK1 was decreased by triptolide in three PCa cell lines, as was the level of P-ULK1 Ser757 (Figure 3B). This indicates that the inhibitory effect of mTOR on ULK1 is suppressed by triptolide, which is consistent with the effect of triptolide on mTOR in PC-3 cells. Furthermore, triptolide increased the level of P-ULK1 Ser317 in three PCa cell lines (Figure 3B), indicating that triptolide may promote phosphorylation of ULK1 by AMPK, thus activating the ULK1 complex. We also examined the effect of triptolide on Beclin 1, an important component of the class III PI3K complex. Activation of Beclin 1 is dependent on phosphorylation at Ser93 and Ser96 by AMPK. We found that although triptolide had different effects on the level of Beclin 1 in three PCa cell lines, it consistently increased the level of P-Beclin 1 Ser93/96 (Figure 3B). This indicates that triptolide also induces activation of the class III PI3K complex.

To determine whether the autophagy induction effect of triptolide is dependent on activation of the ULK1 complex and the class III PI3K complex, we knocked down the expression of ULK1 or Beclin 1 in PC-3 and LNCaP cells with specific siRNAs and examined the level of LC3B-II in the presence or absence of triptolide (50 nM). We found that knockdown of ULK1 (Figure 3C) or Beclin 1 (Figure 3D) reduced the triptolide-induced enhancement of LC3B-II levels. We also used PC-3 cells stably transfected with tandem mCherry-Wassabi-LC3B to investigate the effect of ULK1 or Beclin 1 knockdown on the number of LC3B puncta. Our results showed that knockdown of ULK1 or Beclin 1 decreased the number of LC3B puncta in triptolide-treated cells (Figure 3E). Together, these results demonstrate that triptolide-induced autophagy in PCa cells is dependent on activation of the ULK1 complex and the class III PI3K complex.

### Triptolide induces autophagy by activating AMPK in PCa cells

**Figure 4:**
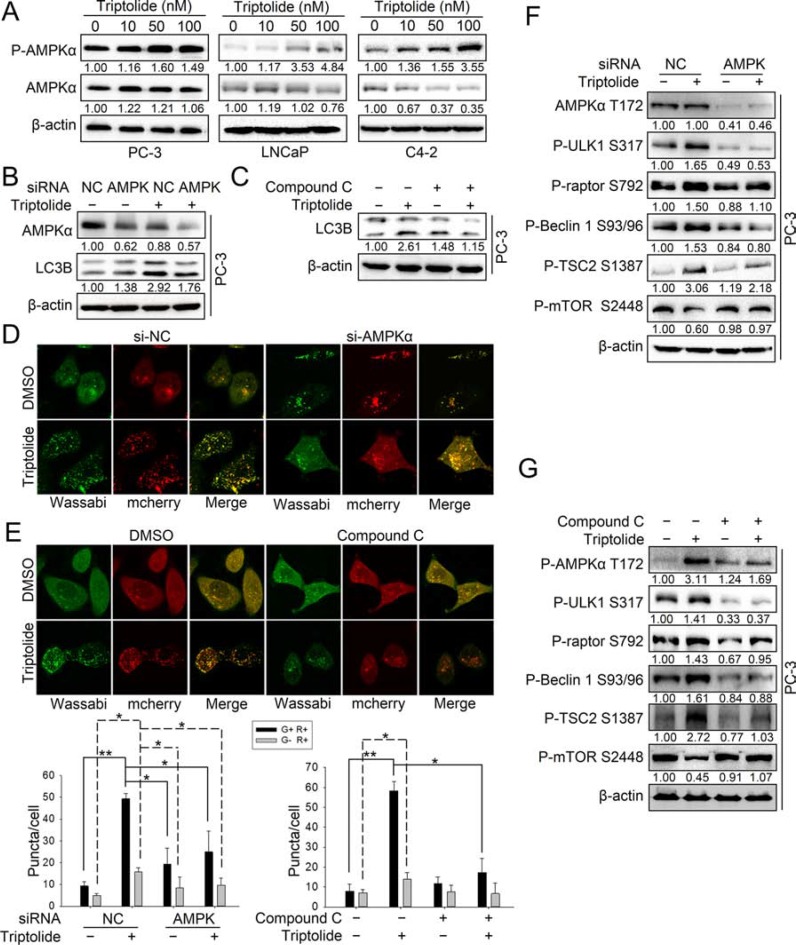
Triptolide activates the AMPK pathway in PCa cells (**A**) PC-3, LNCaP and C4–2 cells were treated with the indicated concentrations of triptolide and incubated for 24 h. Levels of protein expression were analyzed by western blot using antibodies against AMPKα, P-AMPKα Thr172 and β-actin. (**B**) PC-3 cells were co-transfected with AMPKα1 and AMPKα2 siRNAs. 24 h later, cells were treated with 50 nM triptolide or DMSO for another 24 h, and then the samples were subjected to western blot analysis using antibodies against AMPKα, LC3B and β-actin. (**C**) PC-3 cells were pretreated with compound C (10 μM) for 1 h, and treated with 50 nM triptolide or DMSO for 24 h. Samples were subjected to western blot analysis using antibodies against LC3B and β-actin. (**D**) PC-3 cells stably transfected with mCherry-Wassabi-LC3B were further co-transfected with AMPKα1 and AMPKα2 siRNAs and treated with triptolide for 24 h. Cells were then imaged under a confocal microscope. (**E**) PC-3 cells stably transfected with mCherry-Wassabi-LC3B were pre-treated with compound C (10 μM) for 1 h, then co-treated with triptolide for 24 h. The cells were then imaged under a confocal microscope. The average number of G+R+ and G−R+ LC3B puncta per cell from the experiments shown in (D) and (E) are presented in the graphs. (**F**) PC-3 cells were co-transfected with AMPKα1 and AMPKα2 siRNAs and treated with 50 nM triptolide for 24 h. Cell lysates were subjected to western blot analysis using antibodies against the indicated proteins. (**G**) PC-3 cells were pre-treated with compound C (10 μM) for 1 h, then co-treated with 50 nM triptolide for 24 h. Cell lysates were subjected to western blot analysis using antibodies against the indicated proteins. The number under each band represents the fold change of band intensity relative to control group. **P < 0.05. **P < 0.01.

We found that triptolide enhanced the phosphorylation level of several AMPK substrates involved in autophagy activation, including P-raptor Ser792, P-ULK1 Ser317 and P-Beclin 1 Ser93/96. This implies that AMPK may be involved in the induction of autophagy by triptolide in PCa cells. Thus, we investigated the role of AMPK in triptolide-induced autophagy. As shown in Figure 4A, triptolide had a different effect on AMPK protein levels in three PCa cell lines, but increased the level of P-AMPKα Thr172 in all of them. This indicates that the AMPK pathway is activated by triptolide in PCa cells. To examine whether AMPK is involved in triptolide-induced autophagy, we inhibited AMPK using AMPKα siRNA or a widely-used AMPK inhibitor, compound C, in PC-3 cells. The results showed that both siRNA and compound C (10 μM) suppressed the triptolide (50 nM)-induced LC3B II increase (Figure 4B and 4C) and LC3B puncta formation (Figure 4D and 4E). These results indicate that triptolide induces autophagy in PCa cells through AMPK activation.

AMPK promotes autophagy through multiple pathways. AMPK directly phosphorylates ULK1 at multiple sites and Beclin 1 at Ser93/96, leading to autophagy initiation. AMPK phosphorylates TSC2 at Ser1387 and raptor at Ser792 to indirectly inhibit mTOR kinase activity [25]. To examine how AMPK acts in triptolide-induced autophagy, we determined the levels of these phosphorylation substrates of AMPK in PC-3 cells transfected with specific AMPKα siRNAs or treated with compound C (10 μM) in the presence or absence of triptolide (50 nM). The results showed that inhibition of AMPK reduced the levels of P-Beclin 1 Ser93/96, P-ULK1 Ser317, P-raptor Ser792 and P-TSC2 Ser1387 in the presence of triptolide (Figure 4F and 4G). AMPK inhibition rescued the level of P-mTOR Ser2448 (Figure 4F and 4G). These results demonstrate that AMPK activates autophagy through multiple pathways in PCa cells with triptolide treament.

### Triptolide induces autophagy by activating the CaMKKβ-AMPK signaling pathway

**Figure 5:**
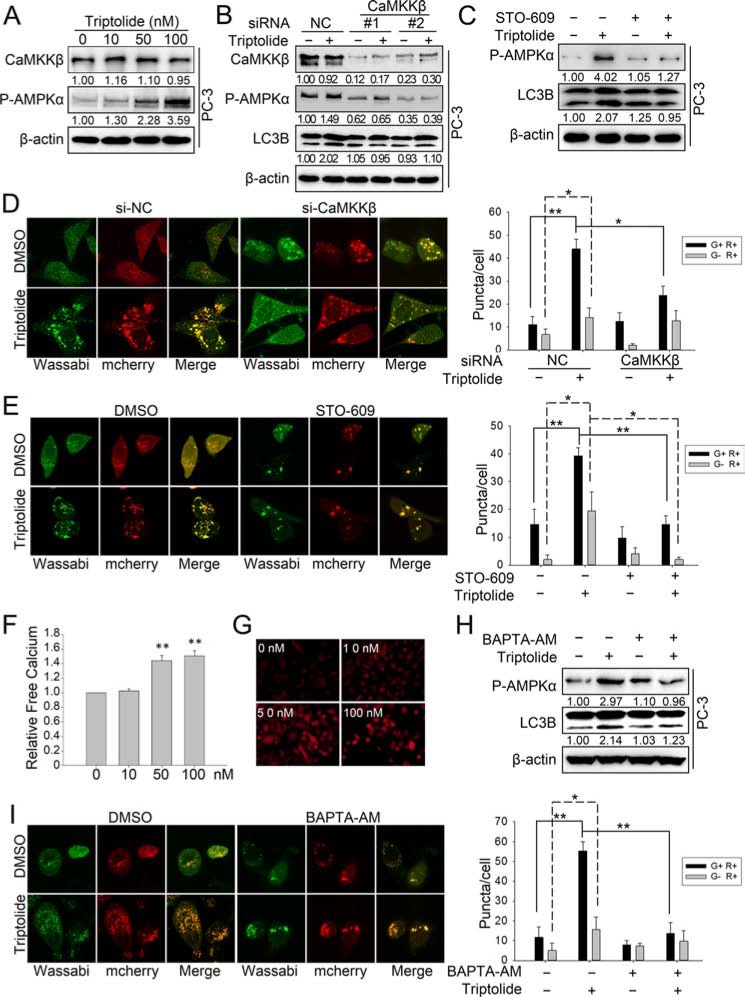
Triptolide induces autophagy by activating the CaMKKβ-AMPK signaling pathway (**A**) PC-3 cells were treated with the indicated concentrations of triptolide and incubated for 24 h. Levels of protein expression were analyzed by western blot using antibodies against CaMKKβ, P-AMPKα Thr172 and β-actin. The number under each band represents the fold change of band intensity relative to control group. (**B**) PC-3 cells were transfected with CaMKKβ siRNA. 24 h later, cells were treated with 50 nM triptolide or DMSO for another 24 h, and then the samples were subjected to western blot analysis using antibodies against CaMKKβ, P-AMPKα Thr172, LC3B and β-actin. (**C**) PC-3 cells were pre-treated with STO-609 (10 μM) for 1 h, then treated with 50 nM triptolide for another 24 h. Levels of protein expression were analyzed by western blot using antibodies against P-AMPKα Thr172, LC3 and β-actin. (**D**) PC-3 cells stably transfected with mCherry-Wassabi-LC3B were further transfected with CaMKKβ siRNAs and treated with 50 nM triptolide for 24 h. Cells were subjected to confocal microscope analysis. The graph shows the average number of G+R+ and G−R+ LC3B puncta per cell. (**E**) PC-3 cells stably transfected with mCherry-Wassabi-LC3B were pre-treated with STO-609 (10 μM) for 1 h, then treated with 50 nM triptolide for 24 h. The cells were then subjected to confocal microscope analysis. The graph shows the average number of G+R+ and G−R+ LC3B puncta per cell. (**F**) and (**G**) PC-3 cells were treated with increasing concentrations of triptolide for 24 h, and the free Ca^2+^ level was measured with Fluo-4 using a microplate reader (F) and a fluorescence microscope (G). (**H**) PC-3 cells were pre-treated with BAPTA-AM (10 μM) for 1 h, then treated with 50 nM triptolide for 24 h. Levels of protein expression were analyzed by western blot using antibodies against P-AMPKα Thr172, LC3B and β-actin. (**I**) PC-3 cells stably transfected with mCherry-Wassabi-LC3B were pre-treated with BAPTA-AM (10 μM) for 1 h, then treated with 50 nM triptolide for 24 h. Cells were then subjected to confocal microscope analysis. The graph shows the average number of G+R+ and G−R+ LC3B puncta per cell. **P < 0.05. **P < 0.01.

We have demonstrated that AMPK activation is important for the autophagy induction effect of triptolide. Next, we wanted to determine the mechanism of triptolide -induced AMPK activation. AMPK is activated by elevation of the AMP: ATP ratio during stress-induced energy deprivation, which is mainly mediated by a serine/threonine kinase, LKB1 [26]. Therefore, we firstly examined the effect of triptolide on LKB1. The result showed that triptolide increased the level of both LKB1 and P-AMPKα Thr172 (Figure S1A). However, knockdown of LKB1 with specific siRNAs had no effect on the the level of P-AMPKα Thr172 or LC3B-II increased by triptolide (Figure S1B). These results demonstrate that LKB1 is not involved in triptolide-induced AMPK activation and autophagy. We further investigated another important AMPK regulator, CaMKKβ, which is a Ca^2+^/calmodulin-dependent serine/threonine kinase [40]. The results showed that triptolide has no effect on the level of CaMKKβ, but increased the level of P-AMPKα Thr172 (Figure 5A). Interestingly, knockdown of CaMKKβ with specific siRNAs or disruption of CaMKKβ activity with the specific inhibitor STO-609 (10 μM) reduced the level of P-AMPKα Thr172 and the formation of LC3B-II in the presence of triptolide (50 nM) (Figure 5B and Figure 5C). Disrupting CaMKKβ activity also decreased the number of LC3B puncta induced by triptolide in PC-3 cells (Figure 5D and 5E). These data demonstrate that triptolide-induced AMPK phosphorylation and activation occurs mainly through CaMKKβ.

It is well known that CaMKKβ is a Ca^2+^-dependent kinase. The kinase activity of CaMKKβ is enhanced by the accumulation of cytoplasmic free calcium [27, 28]. Although triptolide has no significant effect on the CaMKKβ protein level, we reasoned that triptolide may activate CaMKKβ by raising the cellular level of free calcium. To confirm our hypothesis, we used a calcium-specific fluorescent probe, Fluo-4 AM, to measure the free calcium concentration in PC-3 cells treated with triptolide. The result showed that triptolide strongly induces accumulation of cytoplasmic free calcium in a dose-dependent manner (Figure 5F and 5G). To further investigate whether activation of CaMKKβ-AMPK by triptolide is Ca^2+^-dependent, we monitored triptolide-induced AMPK activation and autophagy in cells treated with a calcium chelating agent, BAPTA-AM. Our results showed that chelation of cytoplasmic free calcium by BAPTA-AM (10 μM) antagonized the level of P-AMPKα Thr172 and formation of LC3B-II in the presence of triptolide (50 nM) (Figure 5H). BAPTA-AM also decreased the number of LC3B puncta induced by triptolide in PC-3 cells (Figure 5I). These results indicate that triptolide activates the CaMKKβ-AMPK pathway by inducing accumulation of cytoplasmic free calcium.

### Triptolide induces autophagy via ER stress-dependent calcium release

**Figure 6:**
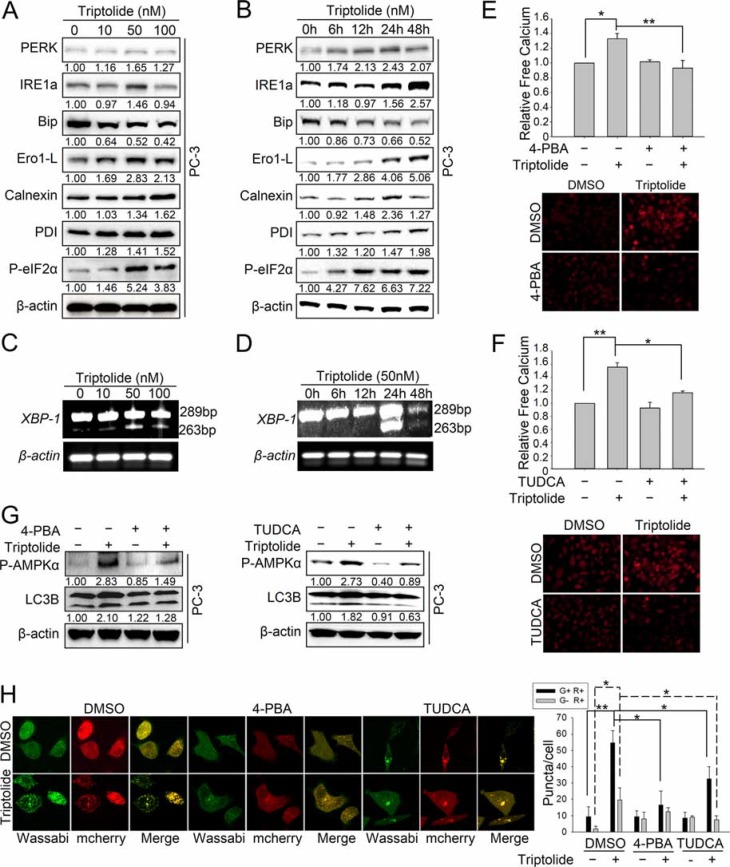
Triptolide induces autophagy via ER stress-dependent calcium release (**A**) PC-3 cells were treated with the indicated concentrations of triptolide and incubated for 24 h. Levels of protein expression were analyzed by western blot using antibodies against indicated ER stress-related proteins and β-actin. The number under each band represents the fold change of band intensity relative to control group. (**B**) PC-3 cells were treated with 50 nM triptolide for the indicated times. Levels of protein expression were analyzed by western blot using antibodies against indicated ER stress-related proteins and β-actin. (**C**) and (**D**) PC-3 cells were incubated with the indicated concentrations of triptolide for 24 h or with 50 nM triptolide for the indicated times. Total RNA was isolated and reverse transcribed. Spliced and unspliced *XBP1* RNAs were amplified by PCR. The products were separated by electrophoresis on a 2.5% agarose gel and visualized by ethidium bromide staining. (**E**) and (**F**) PC-3 cells were pre-treated with 4-PBA (1 mM) or TUDCA (0.5 mM) for 1 h, then incubated with triptolide for another 24 h. Free Ca^2+^ was analyzed with Fluo-4 using a microplate reader and a fluorescence microscope. (**G**) PC-3 cells were pre-treated with 4-PBA (1 mM) or TUDCA (0.5 mM) for 1 h, then incubated with triptolide for another 24 h. Levels of protein expression were analyzed by western blot using antibodies against P-AMPKα Thr172, LC3B and β-actin. (**H**) PC-3 cells stably transfected with mCherry-Wassabi-LC3B were co-treated with 4-PBA (1 mM) or TUDCA (0.5 mM) and triptolide. Cells were then imaged by confocal microscopy. The graph shows the average number of G+R+ and G−R+ LC3B puncta per cell. **P < 0.05. **P < 0.01.

ER stress is one of the signaling pathways involved in regulation of calcium release [27, 29]. It has been reported that triptolide can induce endoplasmic reticulum (ER) stress in cancer cells [30]. Thus we hypothesized that triptolide induces ER stress, causing free calcium to be released from the ER and to accumulate in the cytoplasm. This in turn results in activation of the CaMKKβ-AMPK signaling pathway and autophagy induction. To test our hypothesis, we first examined whether triptolide induces ER stress in PCa cells. As shown in Figure 6A and 6B, the levels of ER stress bio-markers including PERK, IRE1α, P-eIF2α, Ero1-L, calnexin and PDI, were increased by triptolide in both dose-dependent and time-dependent manners in PC-3 cells. Bip, a negative regulator of ER stress, was decreased. Splicing of *XBP1* RNA, which encodes a downstream effector of IRE1α, was also increased by triptolide treatment in PC-3 cells (Figure 6C and 6D). Collectively, these results demonstrate that triptolide induces ER stress through both the PERK-eIF2α arm and the IRE1α-XBP1 arm in PCa cells.

To investigate whether the calcium accumulation results from triptolide-induced ER stress, we treated PC-3 cells with two specific ER stress inhibitors, 4-phenyl butyric acid (4-PBA) and Tauroursodeoxycholic acid (TUDCA). The results showed that inhibition of ER stress by 4-PBA (1 mM) or TUDCA (0.5 mM) strongly inhibited triptolide (50 nM)-induced intracellular calcium accumulation (Figure 6E and 6F). This demonstrates that the accumulation of cytoplasmic free calcium is stimulated by triptolide-induced ER stress in PCa cells. Furthermore, 4-PBA and TUDCA antagonized the level of P-AMPKα Thr172 and formation of autophagosomes (Figure 6G and 6H). Overexpression of AMPKα1 or CaMKKβ rescued TUDCA-antagonized LC3B-II formation with 50 nM triptolide-treament (Figure S2A and S2B). Together, these results indicate that triptolide-induced ER stress results in calcium release, leading to CaMKKβ–AMPK pathway activation and autophagy induction in PCa cells.

### Triptolide induces cytoprotective autophagy in PCa cells

**Figure 7:**
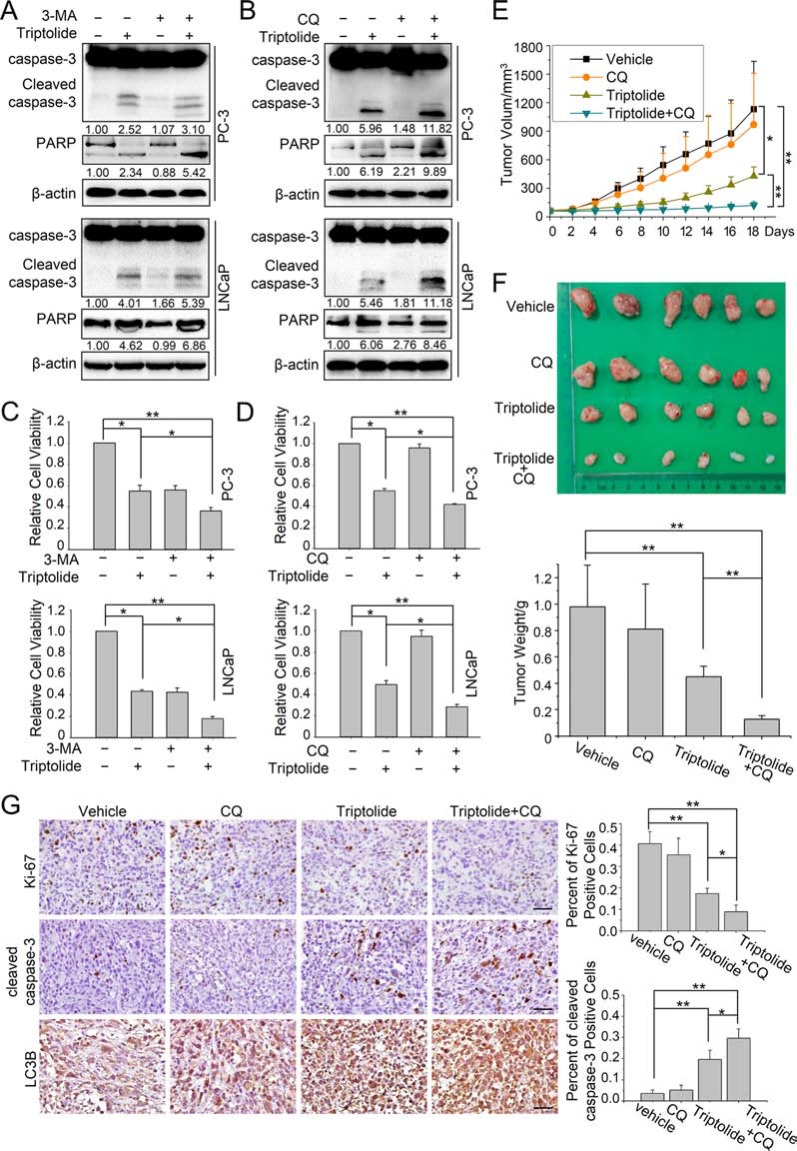
Autophagy plays a protective role in triptolide-treated PCa cells *in vitro* and *in vivo* (**A**) and (**B**) PC-3 and LNCaP cells were pretreated with 3-MA (10 mM) or CQ (3 μM) for 1 h, then incubated with 50 nM triptolide or DMSO for another 24 h. Cell lysates were subjected to western blot analysis using antibodies against caspase-3, PARP and β-actin. The number under the band here represents the fold change of cleaved PARP and cleaved caspase 3 relative to control group. (**C**) and (**D**) PC-3 and LNCaP cells were seeded in 96-well plates (5000 cells per well). After cells had adhered, they were pretreated with 3-MA (10 mM) or CQ (3 μM) for 1 h, then incubated with 50 nM triptolide or DMSO for another 24 h. Cell viability was detected using CCK-8 assays. (**E**) and (**F**) Tumor-bearing nude mice were treated with vehicle, CQ (50 mg/kg/2 days), triptolide (5 mg/kg/2 days) or combined treatment for 18 days. (E) The tumor volume of each group. (F) Image of xenograft tumor and tumor weight of each group. (**G**) Immunohistochemical staining for cleaved caspase-3, Ki-67 and LC3B of tumor form each group. Scale bar: 50 μm. The charts indicate the percentage of Ki-67 positive cells or cleaved caspase-3 positive cells. **P < 0.05. **P < 0.01.

Since autophagy has dual roles in cancer cell survival and cell death, we next investigated the effect of autophagy on the anti-PCa activity of triptolide in PC-3 and LNCaP cells. Firstly we inhibited the triptolide-induced autophagy with 3-MA (10 mM) or CQ (3 μM) and measured triptolide (50 nM)-induced apoptosis by monitoring the level of cleavage of the apoptosis-related proteins caspase-3 and PARP. The results showed that cleavage of caspase-3 and PARP was enhanced in cells co-treated with autophagy inhibitors (3-MA or CQ) and triptolide (Figure 7A and 7B). Furthermore, we examined cell viability to evaluate the effect of autophagy on the cell growth inhibition activity of triptolide (50 nM). The data showed that inhibition of autophagy with 3-MA (10 mM) or CQ (3 μM) enhanced the anti-tumor effect of triptolide (Figure 7C and 7D).

We also tested the effects of autophagy on the anti-PCa activity of triptolide *in vivo*. Nude mice with PC-3 cells tumor xenografts were continuously injected intraperitoneally with PBS, triptolide (0.15 mg/kg/2 days), CQ (50 mg/kg/2 days), and combination of triptolide with CQ until study termination. After the administration of the treatment for 18 days, combined treatment with triptolide and CQ significantly reduced the tumor volume and tumor weight, compared to vehicle treated group and triptolide treated group (Figure 7E and 7F). Meanwhile, the results of immunochemistry assay suggested that the combined treatment increased the level of LC3B and cleaved caspase-3 while reduced Ki-67 expression (Figure 7G). These data suggested that autophagy inhibition also enhanced the cytotoxicity of triptolide in PC-3 cells *in vivo*. Collectively, these results indicate that autophagy protects PCa cells from triptolide-induced cell death.

## DISCUSSION

Triptolide, the major active ingredient of *Tripterygium wilfordii* Hook F., has been shown to have potent anti-tumor activity. Apoptosis was found to be the major mechanism by which triptolide induces cell death [5, 6, 31]. In this study, we demonstrated that triptolide also induces autophagy in human prostate cancer cells. Our results suggested that triptolide stimulates the formation of LC3B-II and increases the number of LC3B-II-positive puncta in three PCa cell lines, PC-3, LNCaP and C4–2. We further found that the triptolide-induced autophagic flux is disrupted by knocking down ATG7, or by using the autophagy inhibitors 3-MA and CQ. Interestingly, blocking the expression of ATG5 significantly inhibited triptolide-induced autophagy in PC-3 cells but not in LNCaP and C4–2 cells. These results imply that triptolide may induce autophagy in LNCaP and C4–2 cells in an ATG5-independent manner. In addition, several other studies have also reported that triptolide induces autophagy in pancreatic cancer, lung cancer and neuroblastoma cells [8, 9, 32]. These findings suggest that induction of autophagy may be a general consequence of triptolide activity.

The serine/threonine kinase mTOR acts as a negative gatekeeper in autophagy regulation. When nutrients are sufficient, the PI3K/Akt pathway activates mTOR through phosphorylation at Ser2448. The activated mTOR inhibits autophagy by phosphorylating and inactivating Atg13 and ULK1, two subunits of the ULK1 complex which are essential for the autophagy initiation progress [23, 33]. During nutrient starvation and stress, the activity of mTOR is inhibited, leading to autophagy induction. Compounds that induce autophagy can be broadly classified into two groups: those that signal via the mTOR pathway (mTOR-dependent), and those that do not (mTOR-independent). For example, rapamycin and its analogs (CCI-779, RAD001, and AP23573) are potent mTOR-dependent inducers of autophagy [34]. They act by stabilizing the raptor-mTOR association by forming a complex with FKBP12, thereby inhibiting the kinase activity of mTOR. Conversely, lithium and L-690, 330 have been reported to induce autophagy by inhibiting inositol monophosphatase rather than by decreasing the kinase activity of mTOR [35, 36]. Our findings showed that triptolide suppresses the level of P-mTOR Ser2448 in PC-3 cells, but has no effect in LNCaP and C4–2 cells. Meanwhile, triptolide increases the level of P-raptor Ser792 in three PCa cell lines. P-raptor Ser792 mediates 14–3–3 binding with mTOR to inhibit mTOR kinase activity. These data suggest that triptolide induces mTOR-dependent autophagy in PC-3 cells, and that triptolide inhibits mTOR activity through direct and indirect mechanisms in these cells.

AMPK acts as an important sensor of intracellular energy levels and is activated in response to metabolic stress to mediate energy balance. Autophagy is one of main mechanisms regulated by AMPK. Our study showed that the AMPK pathway is involved in triptolide-induced autophagy in PCa cells. Triptolide activates AMPK by up-regulating the level of P-AMPKα Thr172, which in turn phosphorylates and activates its substrates, including TSC2, raptor, ULK1 and Beclin 1. This leads to suppression of the negative regulatory effect of mTOR on autophagy and activation of the ULK1 complex and the class III PI3K complex, finally resulting in initiation of autophagy.

AMPK activity is strictly regulated in the cell. The serine/threonine kinase LKB1, a known tumor suppressor, is a key upstream activator of AMPK and phosphorylates AMPK at Thr172 under energy stress conditions [37–39]. Several drugs have been found to induce autophagy through the LKB1-AMPK signaling pathway [40–42]. However, our study showed that LKB1 is not involved in triptolide-induced AMPK activation. Although triptolide enhanced the level of LKB1, knockdown of LKB1 had no effect on the level of P-AMPK Thr172 or LC3B-II following triptolide treatment. Thus, triptolide induced LKB1 accumulation may contribute to the anti-PCa activity of triptolide, but not to the autophagy induction effect. In contrast, we found that CaMKKβ is the key upstream regulator of AMPK following triptolide treatment. CaMKKβ can activate AMPK by phosphorylating Thr172 in response to cellular calcium signaling [28]. In this study, we found that blocking CaMKKβ activity reduces triptolide-induced autophagy and AMPK phosphorylation, which indicates that triptolide induces autophagy through the CaMKKβ-AMPK signaling pathway.

CaMKKβ is a calcium-dependent kinase and its activity is mediated by cellular calcium signaling [28]. In our study, we found that triptolide treatment greatly increased the level of cytoplasmic calcium, which subsequently activated CaMKKβ. Chelation of cytoplasmic free calcium by BAPTA-AM antagonized the triptolide-induced activation of CaMKKβ and induction of autophagy. Krosch et al. also reported that triptolide-induced autophagic cell death in the neuroblastoma cell line SH-SY5Y was accompanied by an increase in intracellular calcium [9]. These findings suggest that the calcium level attributes to triptolide-induced autophagy. As we know, the concentration of cytoplasmic free calcium usually remains very low. In response to certain stimuli, the cytoplasmic calcium level can increase sharply by release from the ER or transport from the extracellular environment [29, 43, 44]. Our data showed that triptolide induces ER stress in PCa cells, and the ER stress inhibitors significantly inhibit the accumulation of cytoplasmic calcium and activation of the CaMKKβ-AMPK signaling pathway in the presence of triptolide. These data demonstrate that the accumulation of cytoplasmic calcium can be mainly attributed to triptolide-induced ER stress. Meanwhile, we found that suppression of ER stress with inhibitors greatly reduced triptolide-induced autophagy. These results indicate that ER stress is critical for triptolide-induced autophagy. It will be worth further examining the relationship between ER stress and triptolide-induced autophagy to investigate whether other mechanisms are involved besides calcium signaling.

Autophagy plays two opposing roles of protector or inhibitor in cell proliferation progress. Our results show that inhibition of autophagy enhances the cytotoxicity and anti-tumor effect of triptolide both *in vitro* and *in vivo*, indicating that triptolide induces cytoprotective autophagy in PCa cells. Recently, it was also reported that autophagy can protect rat cardiomyocytes against triptolide cytotoxicity by preventing apoptosis [45]. However, triptolide was found to induce autophagic death in the metastatic pancreatic cancer cell lines S2–013, S2-VP10 and Hs766T, as well as lung cancer cells A549 and neuroblastoma cells SH-SY5Y [8, 9, 32]. These findings indicate that the effect of autophagy on the anti-apoptosis activity of triptolide is dependent on the cell type.

**Figure 8:**
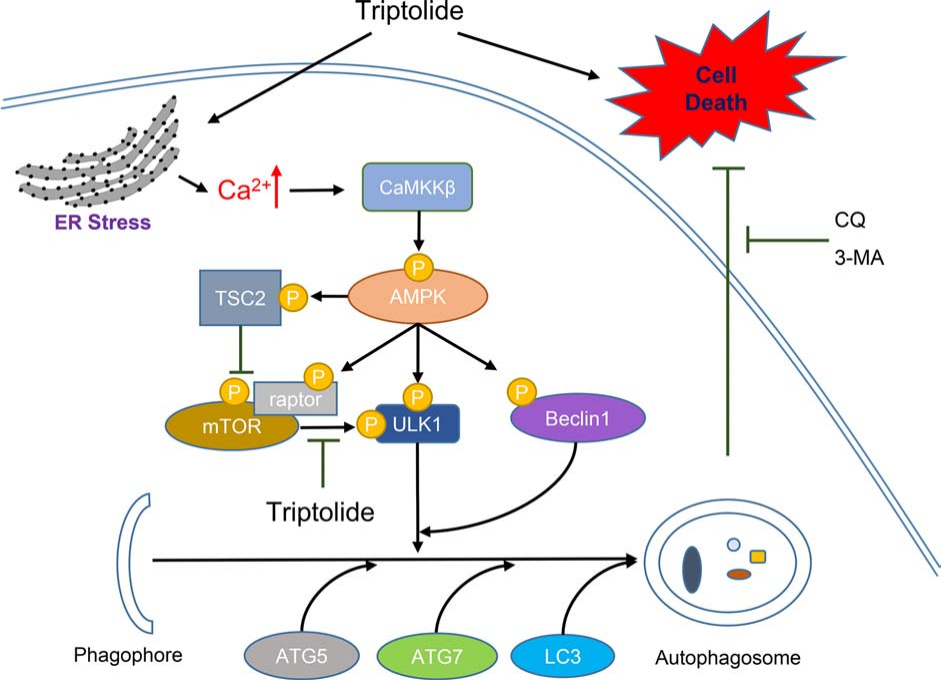
Schematic diagram of hypothetical mechanisms underlying induction of autophagy by triptolide in PCa cells Triptolide induces ER stress, which causes calcium to be released from the ER into the cytoplasm. The high cellular calcium level stimulates CaMKKβ to phosphorylate AMPKα at Thr172. Activated AMPK inhibits mTOR activity through activation of TSC2 and raptor. Inactive mTOR no longer suppresses the ULK1 complex. Activated AMPK also directly phosphorylates ULK1 and Beclin 1, resulting in activation of the ULK1 complex and the Beclin1-VPS34-ATG14L complex. Activation of these complexes leads to the induction of autophagosome formation. Inhibition of autophagy with autophagy inhibitors 3-MA or CQ enhances triptolide-induced cell death in PCa cells.

In conclusion, based on our study, we propose that triptolide induces ER stress, leading to calcium release from the ER. The high cellular calcium level activates CaMKKβ-AMPK signaling pathway, which subsequently induces cytoprotective autophagy in PCa cells (Figure 8). Meanwhile, inhibition of autophagy enhances the anti-PCa effect of triptolide. Thus, we predict that combination treatment with triptolide and pharmacological autophagy inhibitors will be an effective therapeutic strategy for prostate cancer.

## MATERIALS AND METHODS

### Reagents

Triptolide (> 98%) was purchased from π-π Technologies, Inc. and dissolved in DMSO at a stock concentration of 100 mM. Compound C (S7306) and BAPTA-AM (S7534) were from Selleck. 3-Methyladenine (M9281), Chloroquine diphosphate salt (C6628), Sodium tauroursodeoxycholate (T0266) and STO-609 (s1318) were from Sigma. 4-PBA (1821–12–1) was from Meilun Co. Fluo-4 AM Ca^2+^ probe (KGAF024) was from keyGEN BioTECH. Complete Protease Inhibitor Cocktail Tablets (4693116001) and Phosphatase Inhibitor Cocktail Tablets (4906837001) were from Roche. SuperSignal West Pico Chemiluminescent Substrate (34080) was purchased from Thermo Scientific. Lipofectamine 2000 was from Invitrogen. RIPA lysis buffer (P0013B), Caspase-3 antibody (AC031) and PARP antibody (AP102) were from Beyotime Co. Antibodies against mTOR (2983), Phospho-mTOR (Ser2448) (5536), AMPKα (5832P), Phospho-AMPKα (Thr172) (2870), ATG7 (2631S), ATG5 (2630S), ULK1 (8054P), Phospho-ULK1 (Ser317) (6887), Phospho-ULK1 (Ser757) (14202), raptor (2280), Phospho-raptor (Ser792) (2083), Beclin1 (3495P), P-Beclin1 (Ser93/96) (14717) and cleaved caspase-3 (9661) were from Cell Signaling Technology. P62 antibody (sc-28359) was from Santa Cruz. LC3B (L7543) antibody was from Sigma. LKB1 (10746–1-AP) and CaMKKβ (11549–1-AP) antibodies were from Proteintech. Phospho-TSC2 (Ser1387) antibody (AP3338a) was from Abgent. Ki-67 antibody (VP-RM04) was from Vector Laboratories. β-actin antibody (CW0096A) and horseradish peroxidase (HRP)-conjugated goat anti-rabbit and anti-mouse secondary antibodies were from CWBIO. UltraSensitiveTM SP (Rabbit) IHC Kit (KIT-9706) and DAB Detection Kit (DAB-0031) were from Fuzhou Maixin Biotech. Co. pGFPspark-AMPKα1 (HG11488-ACG) plasmid was purchased from Sino Biological Company. GV141-Flag-CaMKKβ (POSE141062597) plasmid was from Shanghai Genechem Company.

### Small interfering RNAs

Gene-specific siRNAs and one non-targeting siRNA were purchased from GenePharma Co. ATG5#1 and ATG5#2 siRNAs target the sequences CCUUUG GCCTAAGAAGAAA and CAUCUGAGCUACCCGG AUA, respectively; ATG7#1 and ATG7#2 siRNAs target the sequences GGAGUCACAGCUCUUCCUU and CAGCUAUUGGAACACUGUA, respectively; Beclin1#1 and Beclin1#2 siRNAs target the sequences GGAA GCUCAGUAUCAGAGA and CAGUUUGGCACAAU CAAUA, respectively; LKB1#1 and LKB1#2 siRNAs target the sequences UGAAAGGGAUGCUUGAGUA and GAAGAAGGAAAUUCAACUA, respectively; AMPKα1 siRNA targets the sequence GAGGAGAGCUAUUUG AUUA; AMPKα2 siRNA targets the sequence GCUGU UUGGUGUAGGUAAA; ULK1 siRNA targets the sequence GCCUGUUCUACGAGAAGAA; while the sequence of the non-targeting control siRNA was 5′-UUCUCCGAACGUGUCACGUTT-3′.

### RNA preparation and *XBP1* splicing assay

Total RNAs were prepared from cultured cells with RNAiso Plus (Takara, 9109). cDNA was reverse transcribed from 1 μg of total RNA with oligo (dT) using a PrimeScript™ RT Master Mix (Takara, R036A). *XBP1* cDNA was amplified by PCR with rTaq DNA polymerase (Takara, R001WZ). The *XBP1* oligonucleotide primers were 5′-TTACGAGAGAAAACTCATGGCC-3′ and 5′-GGGTCCAAGTTGTCCA GAATGC-3′. These primers amplify both the unspliced and spliced human *XBP1* RNAs, yielding products of 289 bp and 263 bp, respectively. The *β-actin* primers were 5′-AATGTCGC GGAGGACTTTGAT-3′ and 5′-AGGATGGCAAGGGAC TTCCTG-3′. The PCR products were separated by electrophoresis on a 2.5% agarose gel (Invitrogen, 75510–019) and visualized by ethidium bromide staining.

### Cell culture and transient transfection

PC-3, LNCaP and C4–2 cells were purchased from the Institute of Basic Medical Sciences, Chinese Academy of Medical Sciences. The cells were cultured in RPMI 1640 medium (GIBCO) supplemented with 10% fetal bovine serum (FBS) and 100 units/ml of penicillin and streptomycin. Cells were incubated at 37°C with 5% CO_2_. Sub-confluent cells with exponential growth were used in all experiments. Transfections were carried out using Lipofectamine 2000 according to the manufacturer's instructions.

### Cell viability assay

5000 cells per well were plated in 96-wells plate, cultured until attached, then treated with various doses of triptolide, using DMSO as the negative control and culture medium as the blank control. 24 h after treatment, 10 μl CCK-8 solution per well was added and the plate was incubated for 1 h at 37°C. The absorbance of each well was measured on an M200pro Multimode Plate Reader (Tecan) at 450 nm and 630 nm. Each treatment was performed in triplicate and experiments were repeated at least 3 times.

### Western blotting

Cell pellets were collected and lysed with RIPA lysis buffer containing 1 × protease inhibitor cocktail and 1 × phosphatase inhibitor cocktail. The protein concentration of cell samples was analyzed using the BCA method. Equal amounts of proteins from each sample were electrophoresed on 8% to 15% SDS-PAGE gels and electrotransferred onto NC membranes. After incubation with appropriate primary and secondary antibodies, proteins were detected using ECL solution and a ChemiDoc XRS+ imaging system (Bio-Rad). β-actin was used as the loading control. The intensity of the bands were analyzed using the ImageJ 1.49 software (National Institutes of Health).

### Tandem mCherry-Wassabi confocal microscopy

PC-3, LNCaP and C4–2 cells stably transfected with mCherry-Wassabi-LC3B were seeded into glass bottom cell culture dishes with 1 × 10^5^ cells per dish. After treatment, the cells were washed with PBS three times and examined under a Nikon A1 confocal microscope system (Nikon, Japan). Images were processed with NIS Element Viewer software (Nikon). Twenty cells were randomly selected to evaluate the average number of mCherry-Wassabi-LC3B puncta per cell.

### Transmission electron microscopy

PC-3 cells were treated with the indicated concentrations of triptolide for 24 h and collected by trypsinization. Cells were fixed with 2.5% phosphate-buffered glutaraldehyde, and post-fixed in 1% phosphate-buffered osmium tetroxide. After being embedded, sectioned, and double-stained with uranyl acetate and lead citrate, photos were taken using an H-7650 transmission electron microscope (Hitachi).

### Free calcium detection assay

PC-3 cells were grown on sterilized glass coverslips overnight and treated with the indicated concentrations of triptolide for 24 h. The medium was then removed and the cells were washed with calcium-free wash buffer (135 mM NaCl, 2 mM KCl, 2 mM MgCl_2_, 10 mM HEPES, 4g/L glucose, pH 7.4). Equal volumes of 1 mM Fluo-4 AM and 20% pluronic F-127 were mixed and added to calcium-free wash buffer to prepare Fluo-4 AM loading solution. The final concentration of Fluo-4 AM was 5 μM. Cells were incubated in the Fluo-4 AM loading solution for 30 min at 37°C before the loading solution was removed. Cells were washed three time with calcium-free wash buffer, then incubated at 37°C for another 30 min. Data were analyzed with a BX51 + DP70 fluorescence microscope (Olympus) or an M200pro Multimode Plate Reader (Tecan).

### Mouse xenograft assay and immunohistochemistry staining

The animal study was approved by the Model Animal Research Center of Nanjing University’ Committee for the Ethical Review of Research and performed in the Model Animal Research Center of Nanjing University. 4-to 5-week old male nude mice were fed in standard environment. Each mouse was inoculated s.c. in the double flanks with 2 × 10^6^ PC-3 cells suspended in 0.1 ml of PBS containing 50% Matrigel (BD Biosciences). When tumors reached 50–100 mm^3^, mice were divided randomly into 4 groups (*n* = 3 per group) and injected with PBS, CQ (50 mg/kg, dissolved in PBS), triptolide (0.15 mg/kg, dissolved in DMSO), or the combination of triptolide (0.15 mg/kg) with CQ (50 mg/kg) intraperitoneally every two days for the duration of treatment. Tumor sizes were measured using calipers and their volumes were calculated using a standard formula: volume = 0.52 × width^2^ × length. Mice were euthanized by day 18 after treatment. Tumors of each group were completely removed, photographed, and fixed in 4% formalin for histologic examination. Immunochemistry assay was performed according to the standard protocol. Photos were taken using a BX51 + DP70 microscope (Olympus). Five pictures were randomly selected to evaluate the average number of DAB positive cells.

### Statistical analysis

Data are expressed as mean ± SD from three or more experiments. Statistical analysis was performed using Student's *t*-test. Differences were considered statistically significant at *p* < 0.05.

## SUPPLEMENTARY MATERIALS AND FIGURES


