# Supplementary material for: Triptolide induces protective autophagy through activation of the CaMKKβ-AMPK signaling pathway in prostate cancer cells
Source: Oncotarget. 2015 Dec 29;7(5):5366–82. doi: 10.18632/oncotarget.6783 (PMC4868692; doi:10.18632/oncotarget.6783)
Supplement: Supplementary file 1 [file oncotarget-07-5366-s001.pdf]

Triptolide induces protective autophagy through activation of the CaMKKβ-AMPK signaling pathway in prostate cancer cells

Supplementary Materials

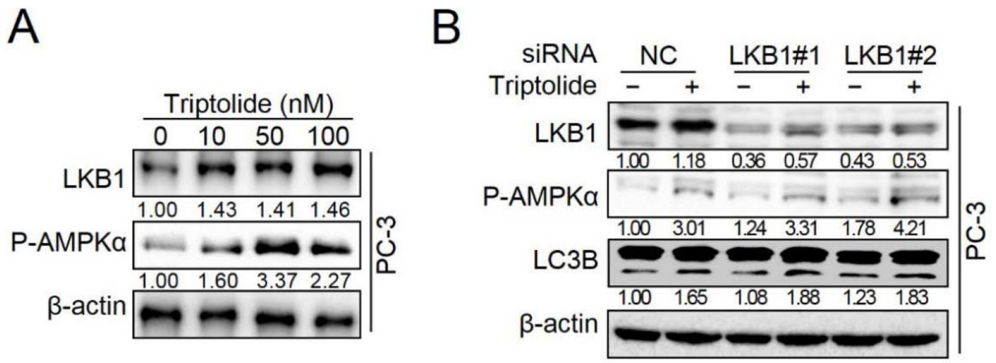

Supplementary Figure S1: Triptolide induces LKB1 up-regulation, but not LKB1-mediated AMPK activation. (**A**) PC-3 cells were incubated with the indicated concentrations of triptolide for 24 h. Levels of protein expression were analyzed by western blot using antibodies against LKB1, P-AMPKα Thr172 and β-actin. (**B**) PC-3 cells were transfected with LKB1 siRNA. 24 h later, cells were treated with 50 nM triptolide or DMSO for another 24 h, and then cell lysates were subjected to western blot analysis using antibodies against LKB1, P-AMPKα Thr172, LC3B and β-actin.

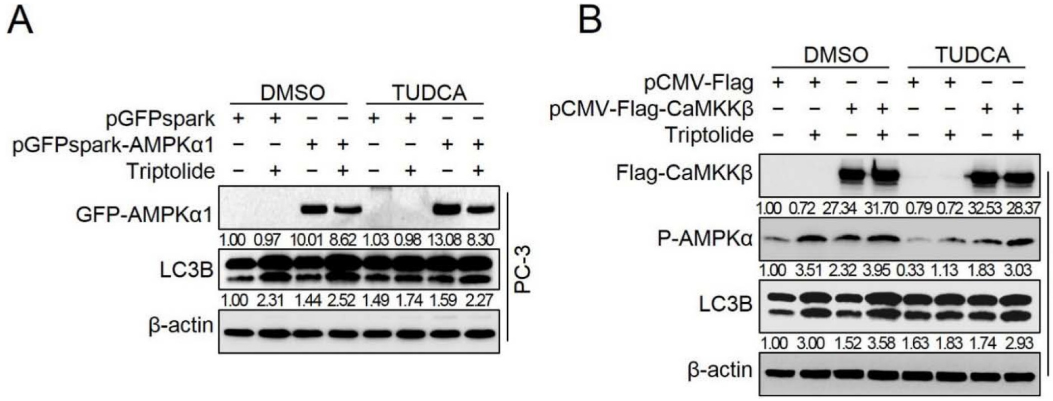

Supplementary Figure S2: Overexpression of AMPKα or CaMKKβ rescues TUDCA induced autophagy inhibition with triptolide treament. (**A**) PC-3 cells were transfected with pGFPspark empty plasmid or pGFPspark-AMPKα plasmid for 24 h, then pretreated with TUDCA (1 mM) for 1 h and treated with 50 nM triptolide for another 24 h. Levels of protein expression were analyzed by western blot using antibodies against GFP, LC3B and β-actin. (**B**) PC-3 cells were transfected with pCMV-Flag empty plasmid or pCMV-Flag-CaMKKβ plasmid for 24 h, then pretreated with TUDCA (1 mM) for 1 h and subsequently treated with 50 nM triptolide for another 24 h. Levels of protein expression were analyzed by western blot using antibodies against Flag, P-AMPKα1 Thr172, LC3B and β-actin.
